# Molecular epidemiology of Newcastle disease virus isolates from vaccinated commercial poultry farms in non-epidemic areas of Japan

**DOI:** 10.1186/1743-422X-10-330

**Published:** 2013-11-09

**Authors:** Dennis Villaseñor Umali, Hiroshi Ito, Terumasa Suzuki, Kazutoshi Shirota, Hiromitsu Katoh, Toshihiro Ito

**Affiliations:** 1 Department of Veterinary Clinical Sciences, College of Veterinary Medicine, University of the Philippines Los Baños, Los Baños, Laguna 4031, Philippines; 2 Avian Zoonoses Research Center, Faculty of Agriculture, Tottori University, 4-101 Koyama, Minami, Tottori 680-8553, Japan; 3 United Graduate School of Veterinary Sciences, Yamaguchi University, Yamaguchi 753-8515, Japan; 4 Poultry Products Quality Control (PPQC Co. Ltd), 125-7 Daiwa Dakeonsen, Nihonmatsu, Fukushima 964-0062, Japan

**Keywords:** Fusion-protein, Japan, Newcastle disease virus, Nucleoprotein, RNA dependent RNA polymerase, Neutralizing epitope variant, Phylogenetic analysis

## Abstract

**Background:**

Newcastle Disease (ND) is a highly contagious and economically devastating disease of poultry. At present, limited molecular epidemiological data are available regarding the causes of ND outbreaks in vaccinated commercial poultry farms. Knowing the genomic characteristics of Newcastle disease virus (NDV) infecting commercial poultry operations in spite of vaccination might give important insights on the infection dynamics of these viruses. In addition, molecular analyses at the subgenotype level and studies on the relationship of Japanese NDVs with other isolates from around the world are lacking. Therefore, in the present study, a molecular epidemiological investigation was conducted to characterize nine NDVs isolated from vaccinated commercial poultry flocks in five different Prefectures in non-epidemic areas of Japan between 1969 and 2002.

**Methods:**

Nucleotide sequencing and phylogenetic studies were performed to characterize the complete fusion (F)-protein gene, 3-prime end of the nucleoprotein (NP)-gene and 5-prime end of the RNA dependent RNA polymerase (L)-gene. Sequence data were compared with 180 NDV strains from GenBank representing different NDV genotypes and subgenotypes from different regions of the world at different time periods. Deduced amino acids were analyzed for homologies, recombination and mutation. Recombination events were estimated using Recombination Detection Program (RDP) version 3.44. Phylogenetic trees were constructed to determine evolutionary relationships among strains.

**Results:**

Mean death time (MDT: 48-56 hr), Intracerebral Pathogenicity Index (ICPI: 1.7-1.9) and deduced amino acid sequences of the F0 proteolytic cleavage site (^112^RRQKR^116^) revealed that all nine field isolates were velogenic. Phylogenetic analysis showed that these isolates could be classified into two genetic lineages and three sublineages namely genotypes VIa (lineage 4a), VId (lineage 4d) and VIId (lineage 5d). No recombination events were observed but a point mutation in one of the neutralizing epitope of the F-protein was identified in the field isolates from Japan.

**Conclusions:**

All field isolates from vaccinated commercial poultry in non-epidemic areas of Japan were part of much bigger outbreaks in provinces and regions and, in some cases, continents. In general, four ND panzootics occurred in Japan and that these outbreaks were mostly characterized by co-circulation of genetically distinct virus lineages due to involvements of infected wild birds. The point mutation identified in the field isolates from Japan may be due to escape from vaccine pressure. The identification of such mutation may be useful for future site-directed mutagenesis to understand the dynamics of NDV infection in vaccinated chickens.

## Background

Newcastle Disease (ND) is a highly contagious and economically devastating disease of poultry. It is caused by the Newcastle disease virus (NDV) [also known as avian paramyxovirus type-1 (APMV-1)] of the genus *Avulavirus* of the family Paramyxoviridae. NDV infects a wide range of domestic and wild bird species worldwide. Among animal viruses, it is one of the biggest contributors of economic losses to the world’s economy [1,2].

NDV is an enveloped, non-segmented, single-stranded, negative-sense RNA virus with a helical morphology. Its genome has six open reading frames (ORF) in the order of 3′-NP-P-M-F-HN-L-5′. These genes encode for the following proteins: nucleoprotein (NP), phosphoprotein (P), matrix protein (M), fusion protein (F), hemagglutinin-neuraminidase (HN) and the RNA dependent RNA polymerase (L) respectively. During P-gene transcription, two additional non-structural proteins, the V and the W proteins, are also generated through RNA editing [3]. Based on genomic size and the nucleotide sequences of the F and L genes, NDV strains can be categorized as class I or class II [1]. Class I NDVs, which have a genomic size of 15,198 nucleotides [4], are occasionally isolated from wild aquatic birds and domestic poultry and are mostly avirulent to chickens. Class II NDVs comprise the majority of virulent NDV strains and some avirulent NDV strains [1]. Class II NDVs are further subdivided into 11 genotypes (I-XI) [1,5-10]. Early sublineages of Class II NDVs that occurred before the 1960s (genotypes I to IV) have a genomic size of 15,186 nucleotides, whereas late Class II NDV sublineages (genotypes VI to XI) have a genomic size of 15,192 nucleotides. Class II NDVs under genotype VI and VII are further subdivided into eight (a-h) subgenotypes [5-10]. Aldous *et al.*[11] proposed the creation of lineages and sublineages in classifying NDVs to make it possible to rapidly type future virus isolates on the basis of their nucleotide sequence and make inferences about their origins. They proposed that NDVs could be divided into six broadly distinct groups (lineages 1 to 6), where lineages 3 and 4 were further subdivided into four sublineages (a to d) and lineage 5 was further subdivided into 5 sublineages (a to e). Genotypes I and II correspond to lineage 1 and 2, while genotype III corresponds to sublineage 3a; genotype IV to sublineage 3b; genotype V to sublineage 3c; genotype VIII to sublineage 3d; genotype VIa and VIe to sublineage 4a; genotypes VIb, VIc and VId to sublineages 4b, 4c and 4d; genotypes VIIa, VIIb, VIIc and VIId to sublineages 5a, 5b, 5c and 5d; 5e to previously characterized genotype VII NDVs from Taiwan and a quarantine isolate in UK that formed a separate cluster from other lineage 5 NDVs; and lineage 6 represents a new NDV genogroup. Recently a novel lineage, provisionally named lineage 7 was reported in West and Central Africa [9].

Different NDV strains vary greatly in pathogenicity [12-14]. NDV isolates can be broadly grouped into five pathotypes on the basis of clinical signs in infected chickens. ND may manifest as viscerotropic velogenic, neurotropic velogenic, mesogenic, lentogenic and asymptomatic enteric [14,15]. Other factors, such as host species, host immune status and age, environmental stress, coinfection with other organisms, viral dose and route of exposure, may also influence the severity of the disease [15].

In Japan, ND was first reported during the first panzootic in the 1930s. This panzootic was caused by a genotype III NDV. After this time, large ND outbreaks were reported to occur until commercial vaccines became available in the late 1960s [16]. Since then, sporadic outbreaks, mostly in small unvaccinated backyard flocks and pet birds, have been reported [15]. In spite of vaccination, few sporadic outbreaks in vaccinated commercial poultry have also been observed [17].

At present, limited molecular epidemiological data are available regarding the causes of ND outbreaks in vaccinated poultry farms. Knowing the molecular characteristics of NDV strains affecting commercial poultry in spite of vaccination might give important insights on the possible origins and genetic nature of these viruses which may help in formulating more effective ND prevention and control strategies. In addition, no studies have been performed yet investigating the classification of Japanese NDVs at the subgenotype level and if recombination events occur in Japanese NDVs. Knowing the subgenotype classification of NDVs and occurrence of recombination events are essential since these may provide a more direct understanding on the epidemiological relationship of Japanese NDVs with other strains from different parts of the world, which may help further elucidate the mechanisms of global and transcontinental dynamics of transmission and spread of this disease. Therefore in the present study, field strains of NDVs with different geographical and temporal distribution patterns that were isolated from vaccinated commercial poultry flocks in non-epidemic areas of Japan were analyzed. Sequence data were extensively compared with 180 NDV strains from different parts of the world from different time periods.

## Results

### Biological and pathotypical characterizations

All suspected NDV isolates yielded a 766-bp product in the nested PCR amplification step. This confirmed that all isolates belong to Avian Paramyxovirus type-1 viruses. All strains exhibited mean death time (MDT) of 48 to 56 hours in embryonated chicken eggs. Intracerebral pathogenicity index (ICPI) values ranged from 1.7 to 1.9 (Table 1). Nucleotide sequence analyses of the variable region of the F-gene (47–421 nt) showed that all isolates had multiple basic amino acids at its F0 proteolytic cleavage site (residues 112–117). Predicted amino acid sequence of the F0 cleavage site was ^112^RRQKR^116^ at the F2 protein and phenylalanine (^116^ F^117^) at the N-terminus of the F1 protein for all strains. These results indicated that all isolates were velogenic.

**Table 1:** Clinical profile and biological characterization of the Japanese field strains

**NDV isolate**	**Host (Approximate age)**	**Region of origin**	**Year isolated**	**Clinical profile of affected flocks**	**ICPI**	**MDT**	**Genotype**
JP/Osaka/2440/69	Layer chickens (80d)	Osaka	1969	Mortality of 60-70%; no detailed history; sample received for diagnosis;	1.8	56 h	VIa
JP/Ibaraki/SM87/87	Layer chicken (180d)	Ibaraki	1987	Mortality less than 3%; 25% drop in egg production; gasping and mild respiratory signs	1.7	56 h	VId

JP/Ibaraki/SG106/99	Layer chicken (700d)	Ibaraki	1999	No detailed history; sample received for diagnosis; necropsy lesions were proventriculitis, petechiae in duodenum and lymphocytic tissues	1.7	48 h	VIId

JP/Chiba/BY103/01	Layer chicken (96d)	Chiba	2001	Mortality around 10%, nervous signs, gasping, leg weakness; twisted neck	1.8	48 h	VIId

JP/Chiba/BY7/02	Layer chicken (110d)	Chiba	2002	Mild respiratory signs; gasping, swollen face, 20% drop in egg production; no significant mortality	1.7	56 h	VIId

JP/Ibaraki/IS5/02	Layer chicken (336d)	Ibaraki	2002	Mild respiratory signs; gasping, seven percent decrease in egg production; no significant mortality	1.8	48 h	VIId

JP/Ibaraki/IS2/02	Layer chicken (14d)	Ibaraki	2002	Severe depression	1.9	48 h	VIId

JP/Fukushima/NYF3/02	Layer chicken (532d)	Fukushima	2002	Mild respiratory signs; gasping, soft-shelled eggs; eight percent decrease in egg production; no significant mortality	1.8	48 h	VIId

JP/Miyagi/AGT/02	Layer chicken (250d)	Miyagi	2002	Mild respiratory signs, gasping; greenish diarrhea; 70% decrease in egg production; no significant mortalities	1.8	48 h	VIId


### Genetic and phylogenetic characterizations

A total of 1662 nucleotides encoding for 553 amino acid residues were identified in the complete coding region of the F-gene of all field strains. Six potential N-glycosylation sites (Asn-X-Ser/Thr where X is any amino acid except proline or aspartic acid) located at positions 85 to 87, 191 to 193, 366 to 368, 447 to 449, 471 to 473 and 541 to 543 were recognized. Twelve cysteine residues located at positions 25, 76, 199, 338, 347, 362, 370, 394, 399, 401, 424 and 523 were identified. Comparison of glycosylation sites and cysteine residues showed no changes in the amino acid sequence in all field strains, which may indicate that these sites were highly conserved.

Analysis of seven neutralizing epitopes located at positions 72, 74, 75, 78, 79, 157 to 171 and 343 of the F-protein showed a K to R amino acid substitution at position 78 in seven of the nine strains (Table 2). These strains were JP/Ibaraki/SG106/99, JP/Ibaraki/IS5/02, JP/Chiba/BY103/01, JP/Ibaraki/IS2/02, JP/Miyagi/AGT/02, JP/Chiba/BY7/02 and JP/Fukushima/NYF-3/02. Furthermore, analysis of amino acid substitutions showed 13 point mutations in the variable region of the F-gene of the Japanese field strains (Table 2). Genotypic and subgenotypic-specific amino acid substitutions were also observed, which were consistent with the proposed theory of NDV evolution as reported previously [18].

**Table 2:** Amino acid substitution in the variable region and neutralizing epitopes of the F-gene sequences of the Japanese field strains

	**Hypervariable region**													**Neutralizing epitopes**						
**Virus**	**4**	**10**	**11**	**13**	**20**	**21**	**27**	**52**	**63**	**78**	**93**	**101**	**121**	**72**	**74**	**75**	**78**	**79**	**157-171**	**343**
Consensus^a^	K	P	A	L	M	L	C	I	V	K	T	R	V	D	E	A	K	A	SIAATNEAVHEVT	L
JP/Osaka/2440/1969	-^b^	-	V	-	-	-	-	-	-	-	-	-	-	-	-	-	-	-	-	-
JP/Ibaraki/SM87/1987	-	L	V	P	T	-	-	-	I	-	S	-	I	-	-	-	-	-	-	-
JP/Ibaraki/SG106/1999	I	-	-	-	-	P	R	V	-	R	-	K	-	-	-	-	R	-	-	-
JP/Chiba/BY103/2001	I	-	-	-	-	P	R	V	-	R	-	K	-	-	-	-	R	-	-	-
JP/Ibaraki/IS5/2002	I	-	-	-	-	P	R	V	-	R	-	K	-	-	-	-	R	-	-	-
JP/Ibaraki/IS2/2002	I	-	-	-	-	P	R	V	-	R	-	K	-	-	-	-	R	-	-	-
JP/Miyagi/AGT/2002	I	-	-	-	-	P	R	V	-	R	-	K	-	-	-	-	R	-	-	-
JP/Chiba/BY7/2002	I	-	-	-	-	P	R	V	-	R	-	K	-	-	-	-	R	-	-	-
JP/Fukushima/NYF3/2002	I	-	-	-	-	P	R	V	-	R	-	K	-	-	-	-	R	-	-	-
JP/Sato/30	R	-	-	-	A	-	H	-	-	-	-	-	I	-	-	-	-	-	n.d.	n.d.
US/B1/47	R	-	-	M	A	-	-	-	-	-	-	-	I	-	-	-	-	-	-	-
LaSota/46	R	-	-	M	A	-	-	-	-	-	-	-	I	-	-	-	-	-	-	-
JP/Ishii/62	R	-	V	-	V	-	-	-	-	-	-	-	I	-	-	-	-	-	-	-

^a^The consensus amino acid sequence was derived from 100–180 velogenic, mesogenic and lentogenic NDV strains from GenBank; ^b^Same as consensus sequence.

**Figure 1:**
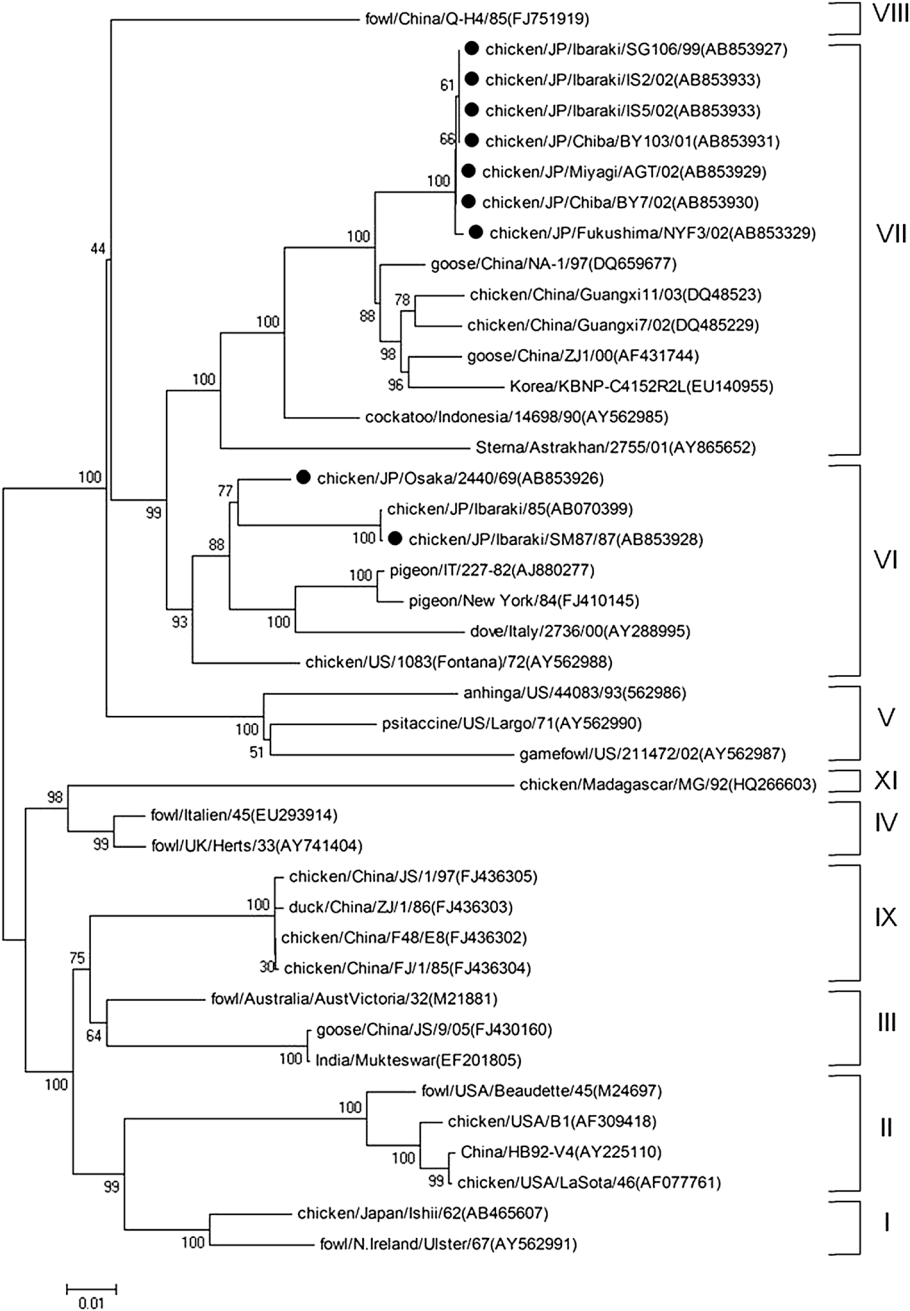
**Phylogenetic tree of the complete coding region of F-gene sequences (1–1662 nt).** The evolutionary history was inferred using the Neighbor-Joining method. The bootstrap consensus tree inferred from 1000 replicates is taken to represent the evolutionary history of the taxa analyzed [40]. Branches corresponding to partitions reproduced in less than 50% bootstrap replicates are collapsed. The percentage of replicate trees in which the associated taxa clustered together in the bootstrap test (1000 replicates) are shown next to the branches. The tree is drawn to scale, with branch lengths in the same units as those of the evolutionary distances used to infer the phylogenetic tree. The evolutionary distances were computed using the Maximum Composite Likelihood [41] method and are in the units of the number of base substitutions per site. All positions containing gaps and missing data were eliminated from the dataset (Complete deletion option). Strains used in this study are marked with •.

**Figure 2:**
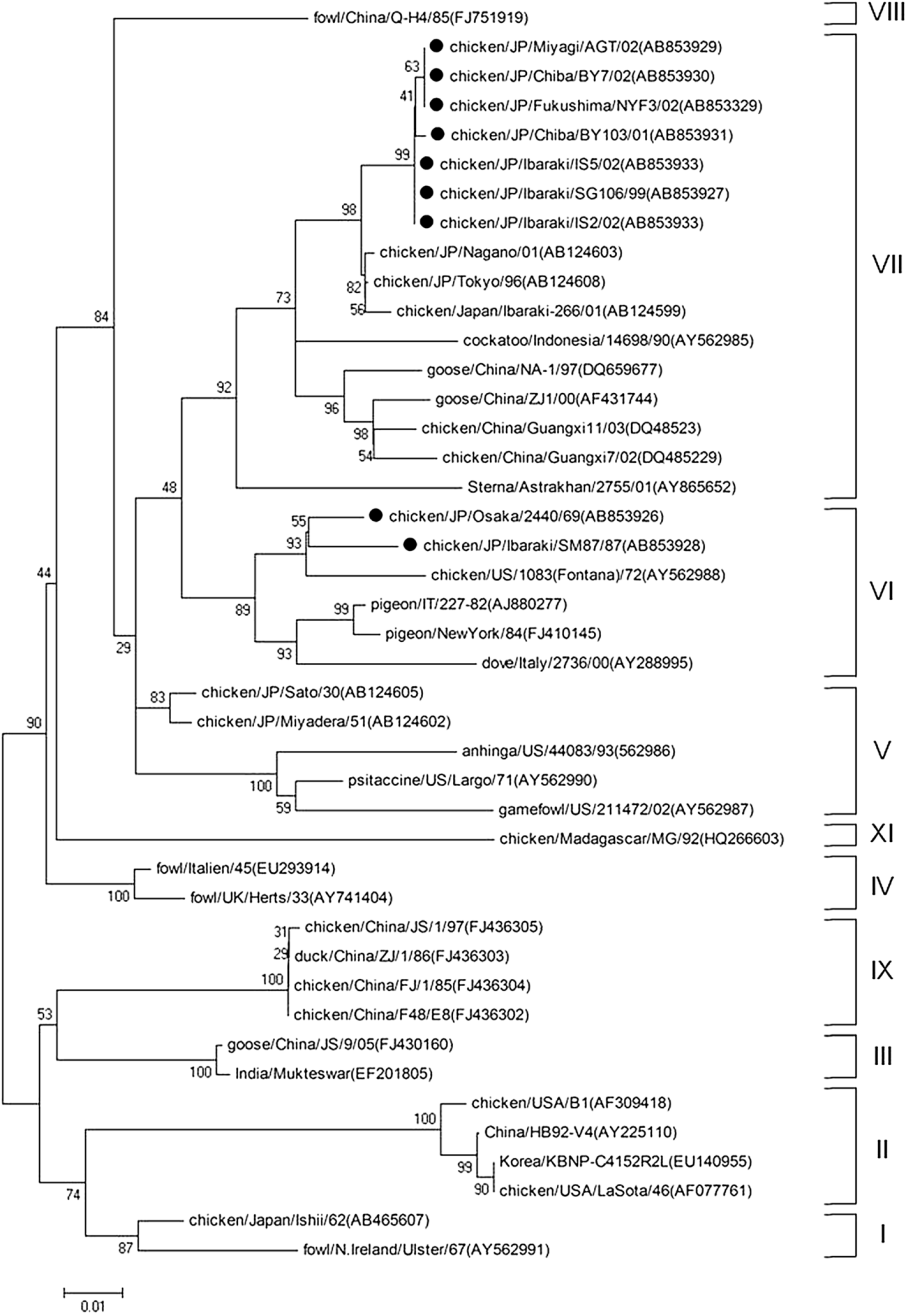
**Phylogenetic tree of the 3-prime end of NP-gene sequences (1–622 nt).** The evolutionary history was inferred using the Neighbor-Joining method. The bootstrap consensus tree inferred from 1000 replicates is taken to represent the evolutionary history of the taxa analyzed [40]. Branches corresponding to partitions reproduced in less than 50% bootstrap replicates are collapsed. The percentage of replicate trees in which the associated taxa clustered together in the bootstrap test (1000 replicates) are shown next to the branches. The tree is drawn to scale, with branch lengths in the same units as those of the evolutionary distances used to infer the phylogenetic tree. The evolutionary distances were computed using the Maximum Composite Likelihood [41] method and are in the units of the number of base substitutions per site. All positions containing gaps and missing data were eliminated from the dataset (Complete deletion option). Strains used in this study are marked with •.

**Figure 3:**
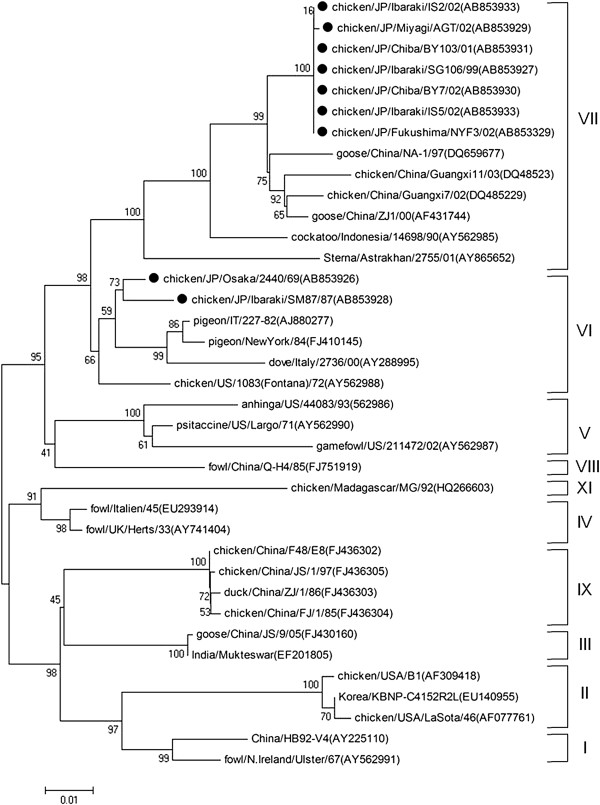
**Phylogenetic tree of the 5-prime end of L-gene sequences (5629–6333 nt).** The evolutionary history was inferred using the Neighbor-Joining method. The bootstrap consensus tree inferred from 1000 replicates is taken to represent the evolutionary history of the taxa analyzed [40]. Branches corresponding to partitions reproduced in less than 50% bootstrap replicates are collapsed. The percentage of replicate trees in which the associated taxa clustered together in the bootstrap test (1000 replicates) are shown next to the branches. The tree is drawn to scale, with branch lengths in the same units as those of the evolutionary distances used to infer the phylogenetic tree. The evolutionary distances were computed using the Maximum Composite Likelihood [41] method and are in the units of the number of base substitutions per site. All positions containing gaps and missing data were eliminated from the dataset (Complete deletion option). Strains used in this study are marked with •.

Phylogenetic analyses of nine field strains and 180 NDV strains from GenBank were performed by using contiguous nucleotide sequences of the F-gene, NP-gene and L-gene. Reference strains from GenBank were selected as representatives of nine of the 11 ND genotypes (genotypes I to XI) representing isolates from different regions of the world (Figures 1, 2 and 3).

**Figure 4:**
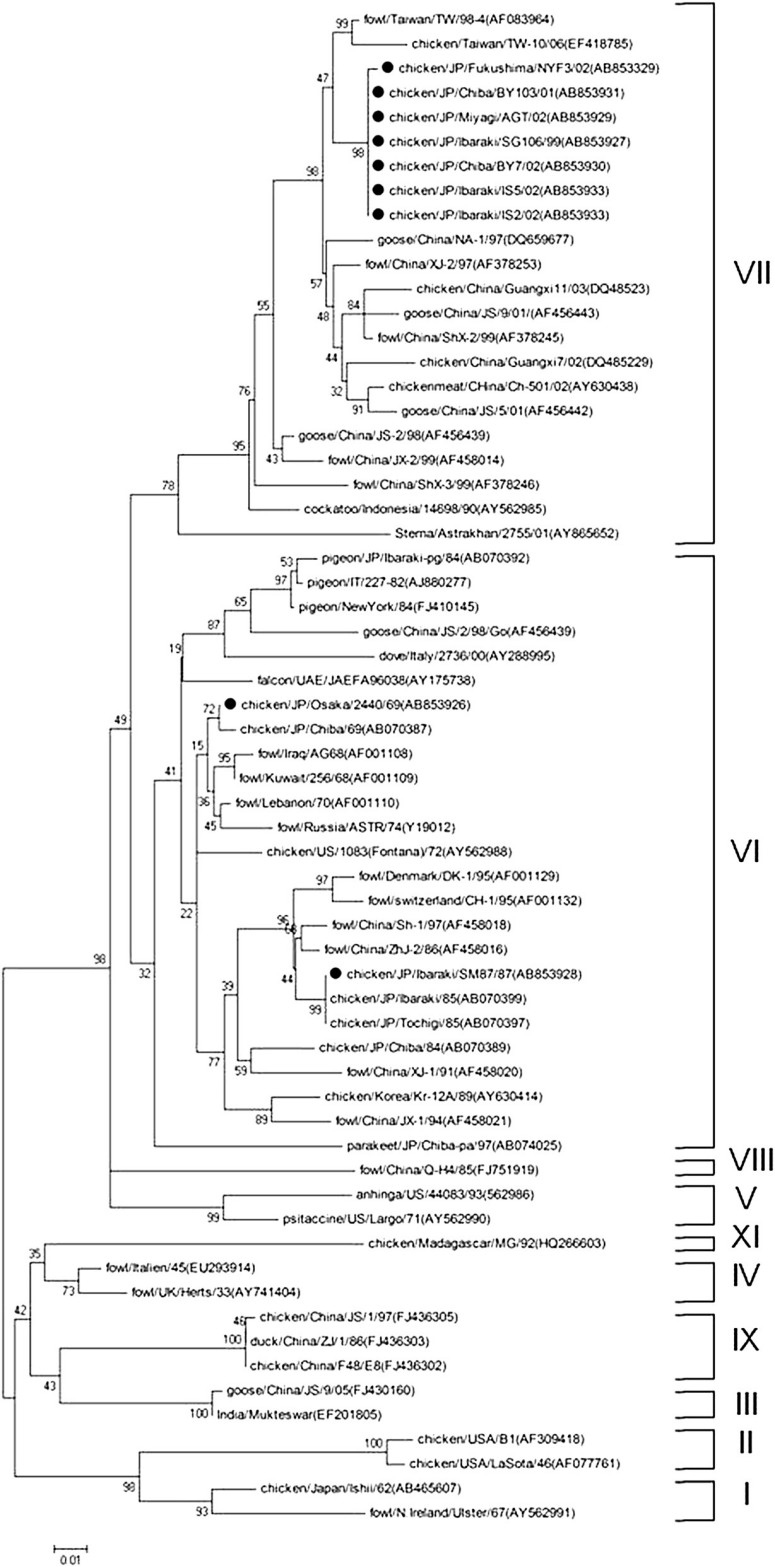
**Phylogenetic tree of the variable region of the F-gene sequences (47–421 nt).** The evolutionary history was inferred using the Neighbor-Joining method. The bootstrap consensus tree inferred from 1000 replicates is taken to represent the evolutionary history of the taxa analyzed [40]. Branches corresponding to partitions reproduced in less than 50% bootstrap replicates are collapsed. The percentage of replicate trees in which the associated taxa clustered together in the bootstrap test (1000 replicates) are shown next to the branches. The tree is drawn to scale, with branch lengths in the same units as those of the evolutionary distances used to infer the phylogenetic tree. The evolutionary distances were computed using the Maximum Composite Likelihood [41] method and are in the units of the number of base substitutions per site. All positions containing gaps and missing data were eliminated from the dataset (Complete deletion option). Strains used in this study are marked with •.

**Figure 5:**
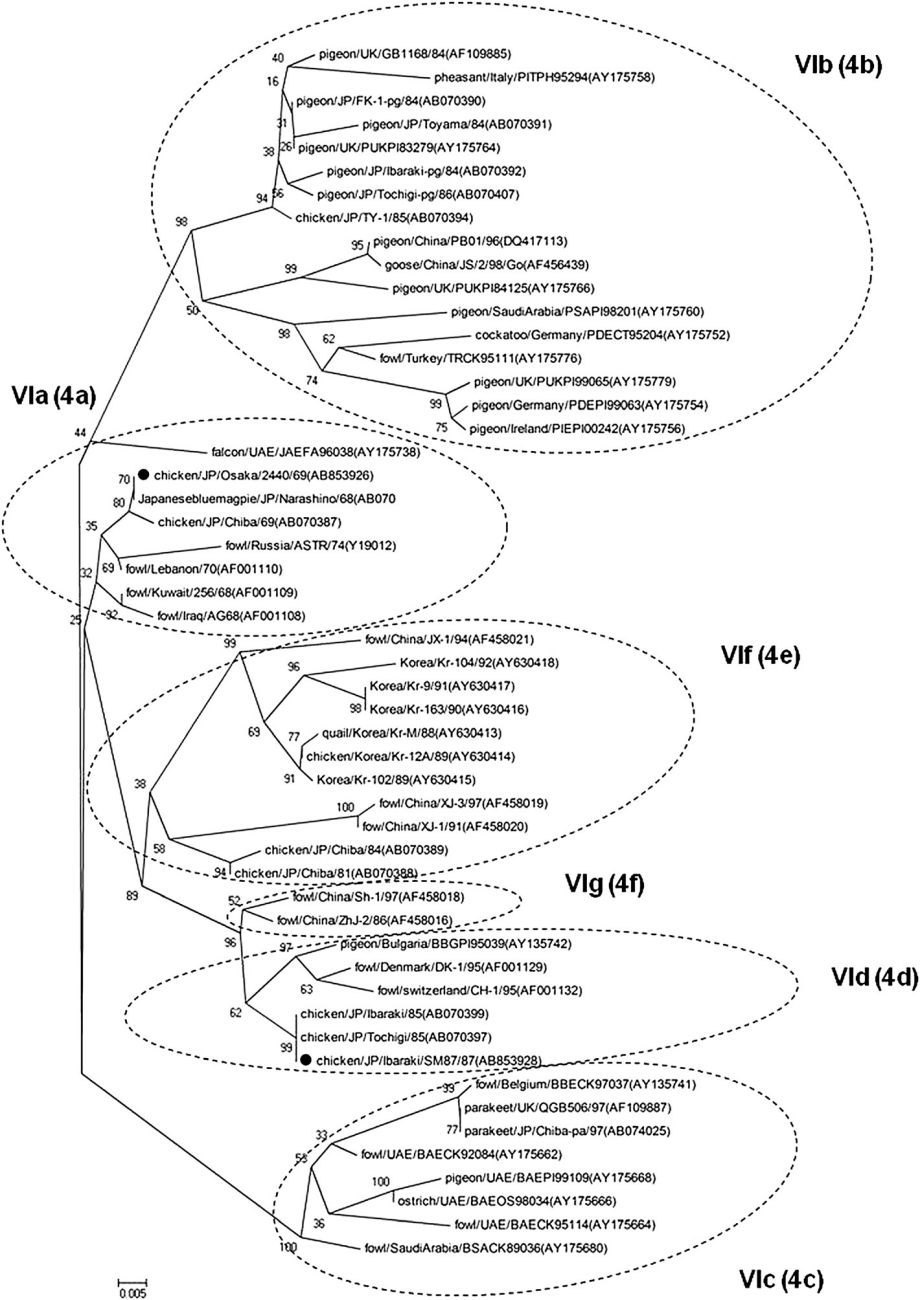
**Straight phylogenetic tree of variable region of the F-gene sequences (47–421 nt) of genotype VI NDV.** The evolutionary history was inferred using the Neighbor-Joining method. The bootstrap consensus tree inferred from 1000 replicates is taken to represent the evolutionary history of the taxa analyzed [40]. Branches corresponding to partitions reproduced in less than 50% bootstrap replicates are collapsed. The percentage of replicate trees in which the associated taxa clustered together in the bootstrap test (1000 replicates) are shown next to the branches. The tree is drawn to scale, with branch lengths in the same units as those of the evolutionary distances used to infer the phylogenetic tree. The evolutionary distances were computed using the Maximum Composite Likelihood [41] method and are in the units of the number of base substitutions per site. All positions containing gaps and missing data were eliminated from the dataset (Complete deletion option). Strains used in this study are marked with •. a-f text inside the parenthesis corresponds to the sublineage grouping.

**Figure 6:**
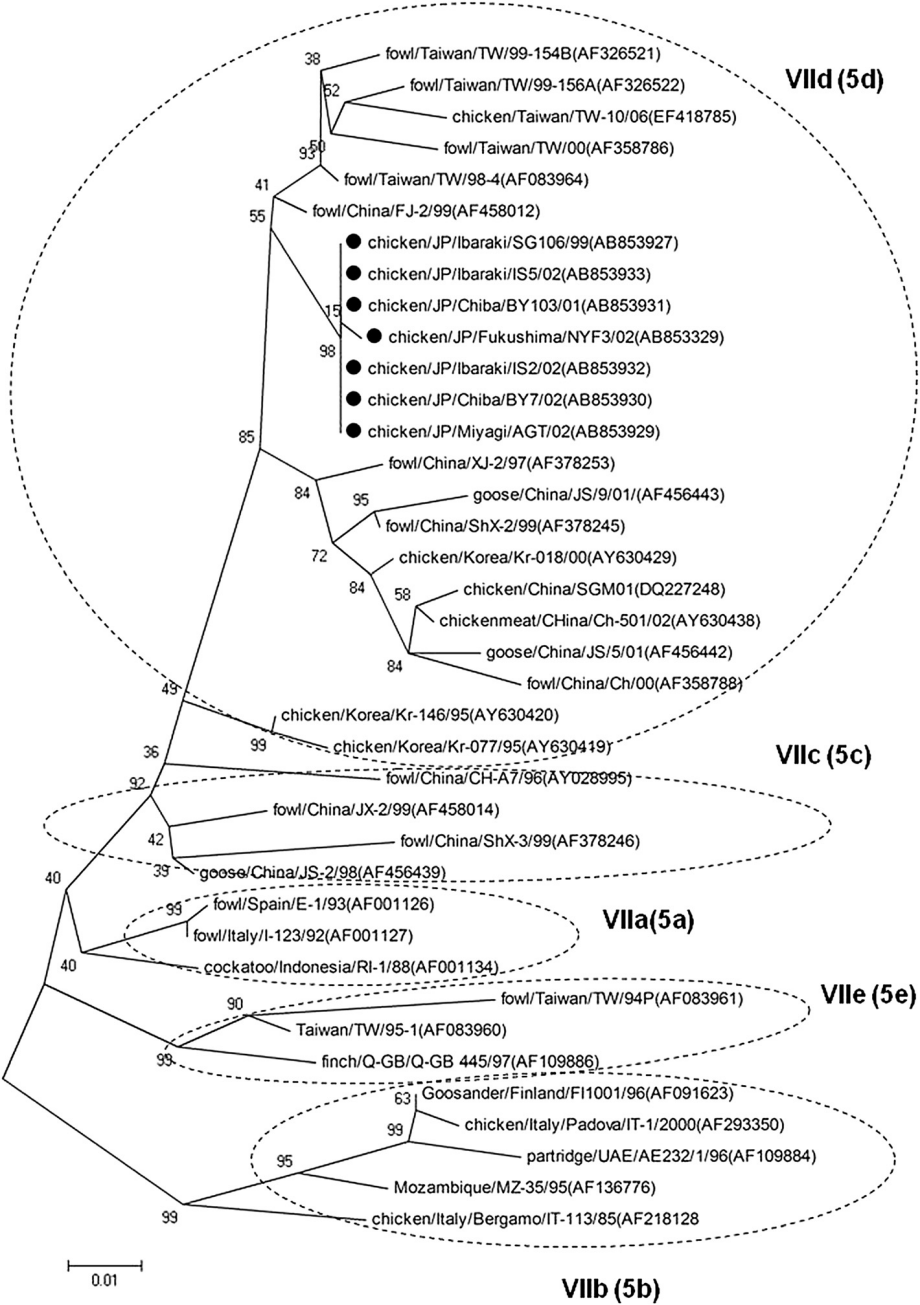
**Straight phylogenetic tree of variable region of the F-gene sequences (47–421 nt) of genotype VII NDV.** The evolutionary history was inferred using the Neighbor-Joining method. The bootstrap consensus tree inferred from 1000 replicates is taken to represent the evolutionary history of the taxa analyzed [40]. Branches corresponding to partitions reproduced in less than 50% bootstrap replicates are collapsed. The percentage of replicate trees in which the associated taxa clustered together in the bootstrap test (1000 replicates) are shown next to the branches. The tree is drawn to scale, with branch lengths in the same units as those of the evolutionary distances used to infer the phylogenetic tree. The evolutionary distances were computed using the Maximum Composite Likelihood [41] method and are in the units of the number of base substitutions per site. All positions containing gaps and missing data were eliminated from the dataset (Complete deletion option). Strains used in this study are marked with •. a-e text inside the parenthesis corresponds to the sublineage grouping.

Field NDV strains were observed to belong to two distinct genotypic groups (genotype VI and VII) using phylogenetic analysis of the complete coding sequence of the F-gene. Early isolates such as JP/Osaka/2440/69 and JP/Ibaraki/SM87/87 were genotype VI, while field strains isolated from 1999 onwards were from genotype VII (Figures 1 and 4). Phylogenetic analysis using the 3-prime portion of NP-gene and 5-prime portion of L-gene yielded the same tree topology and phylogenetic groupings (Figures 2 and 3). Phylogenetic analyses at the subgenotype level using the variable region of the F-gene sequences revealed that JP/Osaka/2440/69 belongs to subgenotype VIa, JP/Ibaraki/SM87/87 to subgenotype VId and all the recent field isolates to subgenotype VIId (Figures 5 and 6).

F-gene nucleotide sequence of JP/Osaka/2440/69 was found to be closely related (98.5-99.2% nucleotide sequence identity) to isolates from the Middle East. The F-gene sequence of JP/Ibaraki/SM87/87 was closely related (96.3-100.0%) with isolates from Japan and China. All the other VIId isolates were highly similar (97.2-100.0%) to isolates from Japan, China and Taiwan and interestingly to a goose isolate from China (98.4-98.6%) (Table 3).

**Table 3:** Nucleotide sequence identity of the field strains using the complete coding region of the F-gene sequences (1–1662 nt)

	**Nucleotide sequence identity (%)**								
**Strain name (genotype)**	**JP/Osaka/2440/69**	**JP/Ibaraki/SM87/87**	**JP/Ibaraki/SG106/99**	**JP/Chiba/BY103/01**	**JP/Chiba/BY7/02**	**JP/Ibaraki/IS-5/02**	**JP/Ibaraki/IS2/02**	**JP/Fukushima/NYF-3/02**	**JP/Miyagi/AGT/02**
JP/Osaka/2440/69 (VIa)	100.0	96.1	92.2	92.2	92.3	92.2	92.2	92.1	92.3
JP/Narashino/68 (VIa)	100.0	95.5	91.4	91.4	91.4	91.4	91.4	91.2	91.4
JP/Chiba/69 (VIa)	99.5	94.9	90.9	90.9	90.9	90.9	90.9	90.6	90.9
Lebanon/70 (VIa)	99.2	95.9	90.8	90.8	90.8	90.8	90.8	90.5	90.8
Kuwait/256/68 (VIa)	99.0	96.1	90.5	90.5	90.5	90.5	90.5	90.2	90.5
Israel/70 (VIa)	98.7	95.9	90.8	90.8	90.8	90.8	90.8	90.5	90.8
Iraq/AG-68 (VIa)	98.5	95.6	90.0	90.0	90.0	90.0	90.0	89.7	90.0
ASTR/74 (VIa)	97.3	94.1	89.3	89.3	89.3	89.3	89.3	89.0	89.3
California/1083(Fontana)/71 (VI)	96.8	94.8	91.9	91.9	91.9	91.9	91.9	91.8	91.9
NewYork/44407/84 (VIb)	95.8	93.9	90.1	90.1	90.2	90.1	90.1	90.1	90.2
JP/Ibaraki/SM87/87 (VId)	96.1	100.0	90.1	90.1	90.2	90.1	90.1	90.0	90.2
JP/Tochigi/85 (VId)	95.5	100.0	88.0	88.0	88.0	88.0	88.0	87.7	88.0
JP/Ibaraki/85 (VId)	96.2	99.9	90.1	90.1	90.2	90.1	90.1	90.0	90.2
Sweden/95 (VId)	95.1	97.6	89.5	89.5	89.6	89.5	89.5	89.4	89.6
DK-1/95 (VId)	94.6	97.3	87.4	87.4	87.4	87.4	87.4	87.1	87.4
CH-1/95 (VId)	94.7	96.8	87.4	87.4	87.4	87.4	87.4	87.2	87.4
TW/99-154 (VId)	94.9	96.3	88.0	88.0	88.0	88.0	88.0	88.2	88.0
Kr-102/89 (VIf)	95.7	95.3	90.3	90.3	90.4	90.3	90.3	90.2	90.4
Zhj-2/86 (VIg)	96.3	98.7	90.2	90.2	90.3	90.2	90.2	90.1	90.3
Sh-1/97 (VIg)	95.8	98.3	89.8	89.8	89.9	89.8	89.8	89.7	89.9
JP/Ibaraki/SG106/99 (VIId)	92.2	90.1	100.0	100.0	99.9	100.0	100.0	99.8	99.9
JP/Chiba/BY103/01 (VIId)	92.2	90.1	100.0	100.0	99.9	100.0	100.0	99.8	99.9
JP/Chiba/BY7/02 (VIId)	92.3	90.2	99.9	99.9	100.0	99.9	99.9	99.8	100.0
JP/Ibaraki/IS-5/02 (VIId)	92.2	90.1	100.0	100.0	99.9	100.0	100.0	99.8	99.9
JP/Ibaraki/IS2/02 (VIId)	92.2	90.1	100.0	100.0	99.9	100.0	100.0	99.8	99.9
JP/Fukushima/NYF-3/02 (VIId)	92.1	90.0	99.8	99.8	99.8	99.8	99.8	100.0	99.8
JP/Miyagi/AGT/02 (VIId)	92.3	90.2	99.9	99.9	100.0	99.9	99.9	99.8	100.0
JP/Ibaraki/00 (VIId)	91.4	88.0	100.0	100.0	100.0	100.0	100.0	99.7	100.0
JP/Gunma/01 (VIId)	91.4	88.0	100.0	100.0	100.0	100.0	100.0	99.7	100.0
FJ-2/99 (VIId)	91.7	88.2	98.7	98.7	98.7	98.7	98.7	98.4	98.7
GD/1/98/Go (VIId)	93.1	91.0	98.5	98.5	98.6	98.5	98.5	98.4	98.6
JP/Tokyo/96 (VIId)	92.0	88.5	98.4	98.4	98.4	98.4	98.4	98.1	98.4
JP/Ibaraki-ph/97 (VIId)	92.0	88.5	98.4	98.4	98.4	98.4	98.4	98.1	98.4
GX-3/98 (VIId)	92.9	90.0	98.3	98.3	98.3	98.3	98.3	97.9	98.3
TW/98-1 (VIId)	91.8	88.4	98.2	98.2	98.2	98.2	98.2	97.9	98.2
TW/98-2 (VIId)	91.5	88.2	98.2	98.2	98.2	98.2	98.2	97.9	98.2
XJ-2/97 (VIId)	93.6	91.2	97.8	97.8	97.9	97.8	97.8	97.7	97.9

No intragenic nor intergenic recombination events (Unique events = 0; Recombination signals = 0) were observed involving the field isolates using all the described methods. Analysis of the over-all mean evolutionary distance among the Japanese field isolates showed rates of 3.9×10^-2^ [standard error (s.e) 0.01], 3.1×10^-2^ (s.e. 0.01) and 2.0×10^-2^ (s.e. 0.01) base substitution per site in the full F-gene, partial NP-gene and partial L-genes, respectively. In contrast, over-all mean evolutionary distance among the recent field strains (1999–2002) were 1.0×10^-3^ (s.e. 0.001) base substitution per site for the F and NP-genes and zero base substitution for the L-gene. In addition, 25 sites in the F-gene of all field strains were observed to be under negative selection (p-value < 0.05) (Table 4).

**Table 4:** **Nucleotide positions in the F-gene sequence of the Japanese field strains that were under negative selection**^**a**^

**Nucleotide position**	**dS**^**b**^	**dN**^**c**^	**dN/dS**	**Normalized dN-dS**	**p-value**
29	70.9	0.0	0.0	−599.4	0.01
80	10.4	0.0	0.0	−88.1	0.04
94	64.8	0.0	0.0	−547.6	0.01
102	32.4	0.0	0.0	−273.7	0.01
106	175.4	0.0	0.0	−1482.8	0.04
109	31.2	0.0	0.0	−263.8	0.03
139	10.5	0.0	0.0	−88.1	0.03
151	196.7	0.0	0.0	−1662.4	0.02
164	10.2	0.0	0.0	−85.9	0.03
173	10.4	0.0	0.0	−88.1	0.04
198	17.6	0.0	0.0	−148.7	0.03
229	33.4	0.0	0.0	−282.5	0.03
230	58.2	0.0	0.0	−491.6	0.02
278	10.4	0.0	0.0	−88.1	0.04
294	17.6	0.0	0.0	−148.7	0.03
295	36.7	0.0	0.0	−310.1	0.03
354	33.4	0.0	0.0	−282.5	0.03
378	86.0	0.0	0.0	−726.7	0.00
405	31.2	0.0	0.0	−263.8	0.03
416	31.2	0.0	0.0	−263.8	0.02
428	215.0	0.0	0.0	−1816.5	0.02
470	58.2	0.0	0.0	−491.7	0.01
474	32.4	0.0	0.0	−273.7	0.01
583	19.8	0.0	0.0	−167.5	0.04
510	33.7	0.0	0.0	−284.4	0.02

^a^ dN < dS negative selection, dN > dS positive selection, dN = dS neutral; ^b^number of synonymous substitutions per synonymous site; ^c^number of non-synonymous substitutions per non-synonymous site.

## Discussion

ND remains a serious threat to commercial poultry even though intensive vaccination programs are being applied. In Japan, occasional outbreaks have been reported in commercial poultry mostly due to improper vaccination, immunosuppression due to infectious and non-infectious causes, and challenge by more velogenic viruses [17]. However, limited data are available regarding the genomic characteristics of NDVs occurring in vaccinated commercial poultry flocks. Knowing the genetic characteristics of wild strains of NDV affecting vaccinated poultry might give important insights on the possible origins, transmission mechanisms and infection routes of these viruses. Molecular and phylogenetic studies like this are important since these might lead to better understanding on how to prevent, control and manage future ND cases.

Molecular characterization of NDV strains mostly considered the F-gene with particular emphasis given on the variable region (47–421 nt) because it codes for a number of functionally important structures such as signal peptide [amino acid (aa) 1–31], cleavage activation sequence (aa 112–116), portion of the fusion inducing hydrophobic region (aa 117–142) and it is characterized by both variable and conserved regions [19,20]. Nucleotide sequence of the F-gene fragment (nt 47–420) is regarded as standard criterion for genotyping [21]. A molecular basis of pathogenicity has also been well established through sequence analysis of F-protein cleavage site. It was reported that the motiff ^112^R/K-R-Q-K/R-R^116^ at the C-terminus of the F2 protein and F (phenylalanine) at the N-terminus of the F1 protein (residue 117) are major determinants of viral virulence [1,2,18,22-25]. A huge database of sequence data especially on F-gene sequences of NDVs isolated throughout the world has also been published and available for sequence comparison and phylogenetic studies [26].

Records of management and farm history showed that NDV strains used in this study originated from farms with diverse geographical, temporal and disease profiles. It is noteworthy to emphasize that these farms were thoroughly vaccinated against NDV but they were still infected with the disease. Moreover, deduced amino acid sequence of cleavage site of the F-gene of all field isolates revealed the motif ^112^R-R-Q-K-R-F^117^ indicating that all strains were velogenic. This was further confirmed by MDT and ICPI tests. These indicate that in spite of the regular use of inactivated and live vaccines, velogenic ND may still occur in vaccinated flocks. However, some affected birds showed only mild respiratory symptoms without significant mortalities and severe pathological lesions. In most flocks, only mild to moderate decrease in egg production was observed.

Seven major epitopes have been identified involving the fusion inhibition and neutralization of F-protein [19,27,28]*.* Individual amino acids at 72, 74, 75, 78, 79 and 343 and a stretch of amino acids from residues 157–171 were identified to be critical for both structures and functions of the F-gene. In this study, nucleotide substitution in one of the fusion inhibition and neutralizing epitope (p.K78R) was identified in all of the seven VIId strains. 13 point mutations were also identified in the variable region of the F-gene. Comparison with sequence data from reference strains (n = 180) showed that among these mutations, p.K4I were conserved only in NDV strains originating from Japan while p.L21P, p.I52V, p.K78R and p.R101K were conserved in strains originating from the Far East Asia (Japan, China and Taiwan). These substitutions maybe used as crude molecular markers of geographic origins of NDVs.

Analysis of genotypic and subgenotypic substitutions in the hypervariable region of the F-gene showed findings that were in conformity with the proposed theory of NDV genetic evolution [18]. It was proposed that subgenotype VIa from the second pandemic probably evolved to VIc by production of a crucial p.S107T substitution and VId by production of p.S93T substitution; VIIb evolved from VIb via a VII-specific p.V121I substitution; VIIb evolved to become VIIa and VIIc through p.K101R substitution; and VIIc evolved to become VIId by the production of additional p.I52V and p.F314Y substitution [18].

A point mutation in the F-gene may have resulted to neutralizing epitope variants. However whether this mutation was part of adaptive mechanism of NDVs to evade the immune response to be able to infect vaccinated chickens is unclear or whether this mutation was actually the effect of selective immune pressure exerted on ND viral particles as a consequence of vaccination is also unknown. To understand how wild NDVs infect vaccinated chickens, this identified mutation may be useful for future site-directed mutagenesis studies.

Phylogenetic analyses on the field strains using the variable region of the F-gene (47–421 nt) revealed that JP/Osaka/2440/69 belongs to genotype VIa (Figure 5). As reported previously [29], genotype VIa was responsible for the second ND panzootic that started in the Middle East during the 1960s and then spread to most countries around the world as a result of enormous trade and importation of captive caged birds and technological advances in air transportation. Interestingly, this isolate shared 100% sequence identity with JP/Narashino/68, which was isolated from a Japanese Blue Magpie. It is interesting to note that nucleotide sequence identity of Iraq/AG-68 was 98.5% similar while strains Kuwait/256/68, Lebanon/70 and Israel/70 were 98.7-99.2% similar with these Japanese strains. It is possible that JP/Narashino/68 was a foreign strain that was introduced to Japan from wild birds. JP/Narashino/68 and/or its progenitor might have been then spread to domestic chickens in Japan, leading to the isolation of JP/Osaka/2440/69 (100% similar) (Table 2).

Phylogenetic analyses on JP/SM87/87 showed that this strain belongs to VId ND viruses (Figure 5). VId viruses together with VIb and VIc were responsible for the third panzootic, which were reported to be spread by pigeons. This strain shared 100% sequence identity with JP/Tochigi/85 and JP/Ibaraki/85, which may indicate that JP/SM87/87 was a product of the ongoing outbreak.

The seven remaining field isolates belong to VIId (Figure 6). Genotype VII is the most predominant NDV genotype that is responsible for most outbreaks in East Asian countries including Taiwan, Korea and China since the 1980s, constituting the fourth pandemic [1,5-7,18,30,31]. Also in Japan, the isolation of genotype VII viruses was reported previously [16]. Therefore, this genotype has been the most predominant NDV in recent outbreaks in Japan.

The earliest VIId viruses on record infected chickens from South Korea in 1995 (Figure 6). These strains include Kr-279/95, Kr-146/95 and Kr-077/95. On the other hand, the earliest VIId NDVs that were reported from Japan were JP/Tokyo/96 from chickens and JP/Ibaraki-ph/97 from a pheasant (99.0% similar to one another), which might indicate that the two strains were part of an ongoing outbreak. Remarkably, JP/Tokyo/96 shared 99.0% sequence identity with GX-1/97, which was isolated from a chicken flock in Western China, FJ-2/99 from a fowl from China and GD/1/98/Go from a goose from China (Table 2). Remarkably, GD/1/98/Go was 98.4-98.6% similar with JP/Ibaraki/SG106/99 and all the other VIId field strains in this study. These findings may indicate that wild birds have played a role in the circulation of VIId viruses across the Far East Asian countries (Korea, Japan and China). A comparison of homologies with contemporary isolates showed that JP/Ibaraki/SG106/99 and all the other VIId field strains were highly similar (99-100%) with JP/Ibaraki/00. Interestingly, a p.K78R amino acid substitution in the F-protein of this strain was reported previously [25]. It was observed that chickens that were challenged with JP/Ibaraki/00 survived a cross-protection test after vaccination with B1 strain, however it was noted that vaccination did not prevent infection and excretion of the virus [25]. This result was partially correlated with the clinical profile of the infected flocks seen in this study. Although the infected flocks survived the infection, problems with production performance were observed.

Comparison among the recent Japanese field strains showed that these strains have high F-gene homologies (99.7-100%), which may indicate that these strains may have been epidemiologically related. Computation of the over-all mean evolutionary distance of the F-gene of these recent strains showed a substitution rate of 1.0×10^-3^ (s.e. 0.001) base substitution per site in the span of 3 years (1999–2002). In contrast, over-all F-gene mean evolutionary distance in all field strains (1969–2002) was 3.9×10^-2^ (s.e. 0.010) base substitution per site. Interestingly, the partial NP gene showed an almost same substitution rate (1.0×10^-3^ and 3.1×10^-2^) while partial L-gene had the lowest rate of substitution (zero and 2.0×10^-2^). However, direct comparison among these substitution rates is not feasible because of incomplete sequence data in NP and L-genes in this study. In other reports, it was shown that among the NDV proteins, the F and P-protein have the highest rate of change (0.78-1.98×10^-3^ and 0.78-2.32×10^-3^ substitution/site/year, respectively) while L and NP-proteins have the lowest rate of change (0.59-1.44×10^-3^ and 0.45-1.50×10^-3^ substitution/site/year, respectively) [32,33]. In addition, analysis of evolutionary selection profiles of the Japanese field strains revealed 25 sites in the F-gene sequence that were under negative selection (p-value < 0.05) (Table 4). No positive selection sites were identified. This is in agreement with the findings of other authors that over-all NDV proteins are under strong purifying and negative selection pressures [32,33].

Phylogenetic analyses using the nucleotide sequences of NP gene, L-gene, complete F-gene coding sequence and variable region of the F-gene resulted to almost similar tree topologies. Surprisingly phylogenetic analysis using NP gene resulted to a clearer differentiation among field strains. These may indicate that NP and L-genes may be alternative methods to characterize NDVs given that like the F-gene, these genes are also involved in the dynamics of viral virulence, play important functional roles in the NDV replication cycle; and are also characterized by regions with high conservation necessary to identify homologies among strains but also characterized by regions with high variations necessary to identify specific variations between strains. Phylogenetic analysis of NP and L-genes in conjunction with the F-gene may also help detect possible natural or artificial recombination events.

## Conclusions

This investigation showed that all field isolates from vaccinated commercial poultry were part of much bigger outbreaks affecting not only provinces or regions but even entire continents. To determine how commercial farms are being infected with NDV, the epidemiology of NDV in the whole of Japan and parts of Far East Asia was analyzed. This study showed that Japanese poultry was affected by at least four pandemics and that outbreaks were mostly characterized by co-circulation of genetically distinct virus lineages that were consistent with the predominant virus genotype circulating in a particular time period. Moreover, no distinct transition was observed from each pandemic. Aside from involvement of local strains, ND outbreaks in Japan were mostly due to virus transmission from infected wild birds either by international bird trade or migration patterns.

A point mutation in one of the neutralizing epitopes of the F-protein resulting to occurrence of neutralizing epitope variants was identified. This identified mutation may be useful for future site-directed mutagenesis to understand the dynamics of NDV infection in vaccinated chickens.

## Materials and methods

### NDV strains

Nine NDV strains isolated from commercial poultry farms with different spatial (Osaka, Ibaraki, Chiba, Fukushima and Miyagi Prefectures) and temporal distribution patterns (1969, 1987, 1999, 2001 and 2002) were used to investigate the molecular epidemiological relationships of ND outbreaks in vaccinated commercial poultry flocks in Japan. These strains were isolated by one to two passages of pooled infected tissues in 10-day-old embryonated specific pathogen-free (SPF) chicken eggs. Infective allantoic fluids were harvested and kept in lyophilized form or in serum tubes and stored at −80°C until further use. All isolates used in this study were provided by Poultry Products Quality Control Co. Ltd. (Fukushima, Japan).

### Farm history and clinical profile

Records of management and farm history were obtained to characterize the clinical profile of nine suspected NDV strains (Table 1). The oldest strain was from Osaka Prefecture in 1969 (JP/Osaka/2440/69). This isolate was recovered from six dead layer birds that were submitted to the Osaka Veterinary Municipal Office for diagnosis. The flock was vaccinated with the live B1 vaccine in drinking water at 5–7 days of age and killed ND vaccine (Sato strain) at 25 days. The affected flock was around 80 days of age when the disease occurred. Mortality was reported to be around 60-70% but no data were given regarding observed clinical signs and production performance. The second strain was from a layer farm in Ibaraki Prefecture isolated in 1987 (JP/Ibaraki/SM87/87). Total population of affected farm was 33,360 birds in 12 open-type houses of 2,780 birds each. The disease was reported in one of the houses. The flock was vaccinated with the live B1 spray at 10 days of age, killed Ishii/B1 at 45 days, live B1 spray again at 60 days and killed Ishii/B1 again at 120 days. The disease occurred two months after the last vaccination at 180 days of age. The disease was characterized by gasping and 25% drop in egg production. Mortality was less than three percent. The third strain was from Ibaraki Prefecture in 1999 that was isolated from dead spent hens sent for diagnosis (JP/Ibaraki/SG106/99). The isolate was from an unknown farm raising spent hens for liquid egg production. No detailed farm information was obtained. Chickens submitted for diagnosis were approximately more than 700 days old. Necropsy findings were proventriculitis, hemorrhagic lesions in duodenum and petechiae in lymphocytic tissues. The fourth strain was from a replacement pullet farm in Chiba Prefecture isolated in 2001 (JP/Chiba/BY103/01). The disease occurred in a flock of 21,000 birds. The flock was vaccinated with the live B1 strain in drinking water at 4 and 10 days of age, live B1 spray at 28 days and killed Ishii strain at 45 and 90 days of age. It was reported that the killed vaccines were injected by hired professional vaccination staff that travel from farm to farms. Six days after last vaccination, the disease occurred characterized by gasping, nervous symptoms, leg weakness, twisting of neck and greenish diarrhea. Mortality was around 10%. The fifth strain was from a layer farm in Ibaraki prefecture isolated in 2002 (JP/Chiba/BY7/02). The affected farm had a population of 125,000 birds (five houses of 25,000 birds) and affected flock was around 25,000 birds (one house). The flock was vaccinated with the live B1 strain in drinking water at 10 days of age, live B1 spray at 24 days, killed Ishii strain at 45 days, live B1 spray again at 60 days and killed Ishii strain again at 95 days. The disease occurred at 110 days of age characterized by gasping, swollen face and infectious bronchitis (IB)-like respiratory signs and 20% drop in egg production without significant mortalities. Necropsy findings were necrotic ovarian follicles and necrotic catarrhal inflammation of the intestines. The sixth strain was from a layer farm located in Ibaraki prefecture in 2002 (JP/Ibaraki/IS5/02). The affected farm had a population of approximately 200,000 birds and the affected flock was around 41,000 birds. The flock was vaccinated with the live B1 spray at 10 and 28 days old, killed Ishii strain at 45 days, live B1 spray again at 60 days and killed Ishii strain again at 90 days. The disease occurred at 336 days of age characterized by mild respiratory signs such as gasping, seven percent decrease in egg production with no marked mortalities. The seventh strain was recovered from dead birds from a replacement pullet farm in Ibaraki prefecture in 2002 (JP/Ibaraki/IS2/02) that were submitted for diagnosis. Total farm population was around 10,000 birds. The flock was vaccinated with the live B1 spray at 10 days of age. The disease occurred four days after vaccination characterized by severe depression. The eight strain was isolated from a layer farm in Fukushima prefecture (JP/Fukushima/NYF-3/02). The farm had a population of around 120,000 birds and affected flock was approximately 16,000 layers. The flock was vaccinated with the live B1 spray at 10 and 28 days of age, killed Ishii strain at 45 days, live B1 spray again at 60 days and killed Ishii strain again at 90 days. The disease occurred around 532 days of age characterized by mild gasping, increase in soft-shelled eggs and eight percent decrease in egg production. Mortalities were minimal and within production standards. The ninth strain was isolated from a layer farm in Miyagi prefecture in 2002 (JP/Miyagi/AGT/02). The flock was vaccinated with the live B1 in drinking water at 10 days of age, live B1 spray at 24 days, killed Ishii strain at 45 days, live B1 spray again at 60 days and killed Ishii strain at 95 days. It was informed that the flock was kept in multiple age houses (six different flocks in one house). Disease occurred at 250 days of age characterized by gasping, greenish white diarrhea and 70% decrease in egg production. No marked mortalities were observed.

### Biological and pathotypical characterizations

Biological and pathotypical characterization of isolates were performed using mean death time (MDT) in 10-days-old embryonated SPF chicken eggs and intracerebral pathogenicity index (ICPI) in 1-day old chicks according to the protocols described in the OIE manual [34]. Confirmation of pathotypes was performed by nucleotide sequence analysis of the F0 proteolytic cleavage site (residues 112–117). Pathotypical data for strain JP/Fukushima/NYF3/02 were obtained in our previous study.

### Reverse transcription polymerase chain reaction (RT-PCR)

Nested RT-PCR was performed to confirm the identities of suspected NDV strains. In brief, isolates were propagated once in 10-day-old embryonated SPF eggs. Viral RNA from infected allantoic fluids was extracted directly by using a QIAamp® Viral RNA Mini Kit (Qiagen, West Sussex, UK). Viral RNA was transcribed to cDNA by using random hexamers and Primescript® Reverse Transcriptase (Takara Bio-Inc, Shiga, Japan). cDNA was amplified by PCR as described previously [16]. A two-step nested PCR was performed to amplify the region comprising the 3′ end of the M-gene and the 5′ end of the F-gene using KOD dash® (Toyobo, Osaka, Japan), 5 uM of external and internal primers as described by Mase *et al.*[16] (Table 5). Thermocycling conditions for the first and second PCR steps were as follows: prewarming at 94°C for 2 min (1 cycle), denaturation at 94°C for 30 sec, annealing at 50°C for 30 sec and extension at 72°C for 1 min. Amplification steps were performed for 35 cycles. The final extension was performed at 72°C for 30 sec (1 cycle).

**Table 5:** Primers used in this study

**Primer name**	**Nucleotide position**^**a**^	**Primer sequence**
NDV-For1	1-15	5′-GGCCACGCGTCGACTAGTACACCAAACAGAGAATC-3′^b^
NDV-For482	482-501	5′-TYTGAGGAGAGRGCACAGAG-3′
NDV-Rev784	803-784	5′-TGYTGTSWRCARAAYTCRTG-3′
M1^c^	4175-4194	5′-TTCTCTAGCAGTGGGACAGC-3′
M2	4241-4264	5′-TGGAGCCAAACCCGCACCTGCGG-3′
F1	5095-5076	5′-CATCTTCCCAACTGCCACTG-3′
F2	5006-4988	5′-GGAGGATGTTGGCAGCATT-3′
NDV-For4359	4359-4382	5′-CCATTGCTAAATACAATCCTTTCA-3′
NDV-Rev4788	4788-4769	5′-GGGGCTTTYGCACACGCCTC-3′
NDV-For4988	4988-5007	5′-AATGCCGCCAACATCCTCCG-3′
NDV-Rev5261	5261-5241	5′-GTGCCTGGATAGTCAGCTGAG-3′
NDV-For5461	5461-5482	5′-GACYTTATCTGTAAGYACAACC-3′
NDV-Rev5731	5731-5711	5′-CAATTGGCAATAACTGAGCC-3′
NDV-For5918	5918-5940	5′-GTGACAGGCAAYCTTGATATATC-3′
NDV-Rev6204	6204-6185	5′-CTTGTAGTGGCTCTCATCTG-3′
NDV-For6369	6369-6388	5′-AGGCYTCACAACATCYGTTC-3′
NDV-Rev6598	6598-6579	5′-TYGATATGCCTRCGAGRTCG-3′
NDV-For13970	13970-13994	5′-GCAGTGGGATATATCACATCTGTGG-3′
NDV-For14492	14492-14511	5′-CAGCCYGTCCGTCCATTCTG-3′
NDV-Rev14739	14739-14720	5′-CTGAGACCCAGTATTGTGAC-3′

^a^The nucleotide position of primers corresponds with the whole genome nucleotide sequences of NDV strain U.S.(CA)/1083(Fontana)/72 (accession # AY562988); ^b^Twenty nucleotide long of 5′-end of primer NDV-For1 is adapter sequence; ^c^M1, M2, F1 and F2 primers are reported by Mase *et al*. [16].

### Nucleotide sequence analysis

Confirmed NDV strains were subjected to additional RT-PCR amplification to characterize the open reading frame of the F-gene sequences (4504–6295 nt of whole NDV genome), 3-prime end of the NP-gene sequences (16–783 nt of whole NDV genome) and 5-prime end of the L-gene sequences (13995–14719 nt of whole NDV genome). In brief, RT-PCR was performed by using SapphireAmp® Fast PCR Master Mix (Takara Bio), 5 uM of forward and reverse primers (Table 5) and cDNAs that were transcribed previously. Thermocycling conditions consisted of initial denaturation at 95°C for 2 min followed by 35 cycles of 98°C for 10 sec (denaturation), 55°C for 10 sec (annealing), 72°C for 10 sec (extensions) and final extension at 72°C for 2 min. PCR products were analyzed by electrophoresis with 1.2% agarose gel and purified by using QIAquick® Gel Extraction Kit (Qiagen, Valencia, CA). The nucleotide sequences of PCR products were determined by Big Dye terminator cycle-sequencing kit version 3.1 (Applied Biosystems Inc., Foster City, CA) and an ABI Prism 3130 Genetic Analyzer (Applied Biosystems). DNA products were sequenced from both directions. The complete F-gene sequence for strain JP/Fukushima/NYF3/02 was obtained in our previous study.

### Phylogenetic studies

Sequence assembly and editing were performed using CodonCode Aligner® (version 3.7.1, CodonCode Corporation, MA) and ClustalX® (version 2.1, Conway Institute UCD Dublin, Ireland). Deduced amino acid sequences were determined using Bioedit® software package version 7.1.3.0 [35]. Confirmation of identity and homology were performed using BLAST http://www.ncbi.nlm.nih.gov.

To determine the molecular epidemiological relationships of field strains, 180 NDV strains isolated from different regions of the world at different time periods were obtained from GenBank. These reference strains were representatives of all the different NDV genotypes and subgenotypes. Phylogenetic and molecular evolutionary analyses were conducted using MEGA version 4 [36]. Phylogenetic trees of the variable region of the F-gene sequences (47–421 nt), complete coding region of the F-gene sequences (1–1662 nt), coding region of the 3-prime end of NP-gene sequences (1–622 nt) and coding region of the 5-prime end of L-gene sequences (5629–6333 nt) were constructed by the neighbor-joining method with the maximum composite likelihood substitution model at 1000 bootstrap replicates.

### Determination of recombination events, evolutionary distances and selection profile

Intragenic recombination events in the NDV nucleotide sequences were determined using RDP v3.44 program. [37]. Seven different algorithms integrated in the program namely RDP, GeneConv, Bootscan, MaxChi, Chimaera, SiScan and 3Seq were applied to detect any putative recombination breakpoints and to estimate the occurrence of any recombination events within all the analyzed genes. Sequences with recombination events identified by at least two detection methods (p < 0.01) were considered as true recombinants. Intragenic recombination events within the F-gene were also determined by comparing the tree topology of phylogenetic analyses using the variable region (47–421 nt) and complete coding region (1–1662 nt) of the F-gene. Intergenic recombination events were determined by comparison of topologies of the generated F, NP and L-gene phylogenetic trees.

Evolutionary distances were calculated using MEGA version 4 using the Maximum Composite Likelihood method [36]. Codon positions included were the 1st + 2nd + 3rd + noncoding. All positions containing gaps and missing data were eliminated from the dataset (complete deletion option). Estimates of s.e. were obtained by a bootstrap procedure of 1000 replicates. Analysis of evolutionary selection profile was performed using Datamonkey http://www.datamonkey.org/ following the Fixed- Effect Likelihood (FEL) method [Hasegawa, Kishino and Yano (HKY) model, p-value less than 0.05] [38,39].

### Accession numbers

Nucleotide sequences used in this study were submitted to the DNA Databse of Japan (DDBJ) with the following accession numbers: JP/Osaka/2440/1969 F-gene sequence: AB853926; NP-gene: AB854728; L-gene: AB854729. JP/Ibaraki/SM87/1987 F-gene sequence: AB853928; NP-gene: AB854730; L-gene: AB854731. JP/Ibaraki/SG106/1999 F-gene sequence: AB853927; NP-gene: AB854732; L-gene: AB854733. JP/Ibaraki/BY103/2001 F-gene sequence: AB853931; NP-gene: AB854734; L-gene: AB854735. JP/Ibaraki/BY7/2002 F-gene sequence: AB853930; NP-gene: AB854736; L-gene: AB854737; JP/Ibaraki/IS5/2002 F-gene sequence: AB853933; NP-gene: AB854744; L-gene: AB854745. JP/Ibaraki/IS2/2002 F-gene sequence: AB853932; NP-gene: AB854742; L-gene: AB854743; JP/Fukushima/NYF-3/2002 F-gene sequence: AB853329; NP-gene: AB854740; L-gene: AB854741; JP/Miyagi/AGT/2002 F-gene sequence: AB853929; NP-gene: AB854738; L-gene: AB854739.

### Ethical approval

Animal experiments in this manuscript were critically reviewed and approved by the Animal Ethical Committee of Poultry Products Quality Control (Permit number: 2013-03A) and performed in accordance with the Japanese laws and international guidelines for the use of animals in research.

## Abbreviations

APMV-1: Avian paramyxovirus type-1; cDNA: Complementary deoxyribonucleic acid; C-terminal: Carboxyl-terminal; F-gene: Fusion gene; FEL: Fixed- effect likelihood; HKY: Hasegawa, Kishino and Yano; ICPI: Intracerebral pathogenicity index; JP/Chiba/BY103/01: APMV1/chicken/Japan/Chiba/BY103/2001; JP/Chiba/BY7/02: PMV1/chicken/Japan/Chiba/BY7/2002; JP/Fukushima/NYF-3/02: APMV1/chicken/Japan/Fukushima/NYF-3/2002; JP/Ibaraki/IS2/02: APMV1/chicken/Japan/Ibaraki/IS2/2002; JP/Ibaraki/IS5/02: APMV1/chicken/Japan/Ibaraki/IS5/2002, JP/Ibaraki/SG106/99, APMV1/chicken/Japan/Ibaraki/SG106/1999; JP/Ibaraki/SM87/87: APMV1/chicken/Japan/Ibaraki/SM87/1987; JP/Miyagi/AGT/02: APMV1/chicken/Japan/Miyagi/AGT/2002; JP/Osaka/2440/69: APMV1/chicken/Japan/Osaka/2440/1969; L-gene: RNA dependent RNA polymerase; MDT: Mean death time; M-gene: Matrix gene; N-terminal: Amino-terminal; ND: Newcastle disease; NDV: Newcastle disease virus; NP-gene: Nucleoprotein gene; nt: Nucleotides; RDP: Recombination detection program; RT-PCR: Reverse transcription polymerase chain reaction; s.e.: Standard error; SPF: Specific pathogen free.

## Competing interests

The authors declare that they have no competing interests.

## Authors’ contributions

HK, TI and HI conceived and designed the research. HI and DVU performed RT-PCR, nucleotide sequencing and generation of phylogenetic trees. HK, TI, HI and DVU analyzed the data: HK, KS and TS collected and provided the NDV samples: HK, TI, HI and DVU drafted the manuscript. All authors’ read and approved the final manuscript.
